# Axillary nodal metastasis in ovarian cancer: a report of three cases and review of literature

**DOI:** 10.1186/s43046-019-0008-x

**Published:** 2019-12-23

**Authors:** Mohammad Zuhdy, Reham Alghandour, Gena Abdelazeem, Gehad A. Saleh, Mahmoud M. Saleh, Mohamed Hamdy, Omar Hamdy

**Affiliations:** 1 Surgical Oncology Unit, Oncology Center, Mansoura University, Mansoura, 35516 Egypt; 2 Medical Oncology Unit, Oncology Center, Mansoura University, Mansoura, Egypt; 3 Pathology Department, Faculty of Medicine, Mansoura University, Mansoura, Egypt; 4 Radiology Department, Faculty of Medicine, Mansoura University, Mansoura, Egypt

**Keywords:** Ovarian cancer, Axillary metastasis, Case report, Neoplasm, Metastasis

## Abstract

**Background:**

Ovarian cancer represents a major global health burden that is rarely associated with distant metastasis. Axillary lymph node metastasis from ovarian cancer is rare and is reported only in few case reports in literature.

**Case presentation:**

We report three cases of ovarian carcinoma associated with axillary lymph node metastasis as well as a brief literature review. The pathologic subtype in one case was malignant mixed Mullerian tumor, while the other two cases were high-grade serous ovarian carcinoma. Axillary nodal metastasis was reported as a synchronous event in one case, while it was reported as recurrence events in the other two cases.

**Conclusion:**

Physicians should be aware of this uncommon mode of metastasis in ovarian cancer cases. Multi-disciplinary discussion is crucial in the management of these cases to offer them the best suitable treatment.

## Background

Ovarian cancer represents a major global health burden accounting for 3.4% of newly diagnosed cancer cases and 4.4% of cancer mortality among females, where most patients die within 5 years of initial diagnosis [1, 2]. This could be attributed to the advanced disease stage at which most patients are presented that makes treatment strategies less efficient [3]. Ovarian cancer mainly metastasizes through intraperitoneal route either to Douglas pouch, paracolic gutters, liver and splenic capsules, omentum, or small intestinal mesentery where it remains confined throughout the disease course [4]. Distant metastasis from ovarian cancer is a rare event where the liver, lung, or pleura are most commonly involved [5]. Axillary lymph node metastasis from ovarian cancer is rare [6] and is reported only in few case reports in literature. Hereby, we present three cases of ovarian serous carcinoma associated with axillary lymph node metastasis as well as a brief literature review.

## Case presentation

### Case no. I

A 69-year-old lady without a relevant medical history complaining of vague abdominal symptoms. Abdominal ultrasonography and computerized tomography (CT) scan revealed bilateral adnexal complex lesions with para-aortic lymphadenopathy, omental, peritoneal deposits, and two hypodense hepatic focal lesions, serum cancer antigen 125 (CA 125) was markedly elevated 990 u/ml (normal limit up to 35 u/ml). Core needle biopsy from the omental lesion revealed metastasis from ovarian carcinoma confirmed by positive immune histochemical (IHC) staining for cytokeratin 7 (CK7), Wilms’ Tumor-1 (WT-1), and negative CK20. Our tumor board recommended neoadjuvant chemotherapy, so the patient received 3 cycles of neoadjuvant (Taxol/carboplatin) regimen. Re-evaluation CT revealed the disappearance of the hepatic and peritoneal lesions, so interval suboptimal debulking was performed in January 2014 in the form of total abdominal hysterectomy, bilateral salping-oophrectomy, iliac lymph node sampling, and infracolic omentectomy with gross residue left in the pelvis to avoid the risk of injury of the left ureter. Postoperative pathology revealed bilateral ovarian infiltration by mixed malignant tumoral growth formed of carcinomatous elements of papillary serous type admixed with sarcomatous area of chondrosarcomatous type surrounded by desmoplastic stroma. Positive cytoplasmic staining of CK7 and positive nuclear staining of WT-1 confirmed the diagnosis of bilateral malignant mixed Mullerian tumor. Iliac lymph nodes were free from tumor tissue while the infracolic omentum was infiltrated (FIGO stage IIIB). The patient then received 6 cycles of adjuvant chemotherapy regimen (Taxol/carboplatin), then she was maintained on follow-up till May 2016 when a follow-up CT scan revealed suspicious right-sided axillary lymph node enlargement. Magnetic resonance imaging of both breasts was free except for the previously reported axillary lymphadenopathy. Axillary lymph node excision biopsy was done, and histopathological examination revealed metastatic carcinoma of ovarian origin positive for IHC CK7 and WT-1 and negative for mammoglobin, ER, and PR. The patient received 6 cycles of Taxol/carboplatin chemotherapy protocol, and post-treatment evaluation CT was free. She was maintained on follow-up with the medical oncology clinic at our center till March 2018 when rising level of CA-125 was detected. CT scan revealed multiple peritoneal nodules scattered in the abdomen and pelvis. That is why she restarted chemotherapy with 6 cycles of Taxol/carboplatin regimen but with a stationary disease course, so she was shifted to Taxol/Gemzar for 3 cycles followed by aromatase inhibitor with stable disease status till her last visit on 10 July 2019.

### Case no. II

A 39-year-old lady with a history of previous right oophorectomy due to ruptured ectopic pregnancy. She was referred to our center in December 2016 after performing left ovarian cystectomy with a postoperative pathology report that revealed papillary serous cystadenocarcinoma. Preoperative MRI revealed multilocular ovarian cystic lesion about 6.5 × 7 × 8 cm with mural nodules, septae, and central solid component. It was associated with minimal free fluid in Douglas pouch as well as hemorrhagic peritoneal nodules. Serum CA 125 was mildly elevated (CA 125 = 87). General examination revealed enlarged right-sided axillary lymph node, so a bilateral breast and axilla sonomammography was requested but it revealed no breast lesions except for the enlarged lymph node from which a fine needle aspiration cytology revealed metastatic papillary adenocarcinoma of ovarian origin confirmed by the positivity of CK7 and WT-1 and negativity of CK20 and mammoglobin. The tumor board recommended proceeding to cytoreductive surgery where total abdominal hysterectomy, left salping-oophrectomy, infracolic omentectomy, excision of peritoneal nodules over the diaphragm and in the pelvis, and right-sided axillary clearance were done. The rationale for performing axillary clearance rather than axillary lymph node excision was to accomplish complete cytoreduction through minimizing the incidence of leaving micrometastasis in any unexcised axillary nodes. Histopathological examination of the specimen revealed high-grade papillary serous cystadenocarcinoma with deposits in the omentum, the peritoneum, and one out of 17 dissected axillary lymph nodes. The patient had a smooth postoperative course then she was referred to a medical oncologist to receive her adjuvant therapy, after which she lost follow-up with our department.

### Case no. III

A 58-year-old postmenopausal lady with a medical history of hypertension and heart failure was presented in April 2016 with a complaint of abdominal enlargement and vague abdominal pain. CT scan revealed large right side malignant adnexal mass 14 × 12 × 10 cm with moderate ascites, enlarged iliac and paraortic lymph nodes, and large peritoneal nodules. This scan also revealed multiple enlarged axillary lymph nodes. Serum CA-125 was markedly elevated 720 u/ml (normal range = up to 35 u/ml), while bilateral sonomammography did not reveal any suspicious lesions. A fine needle aspiration cytology (FNAC) from right-sided enlarged axillary lymph nodes revealed metastatic carcinoma of ovarian origin confirmed by positive WT-1 and negative CK7, CK20, inhibin, and leukocyte common antigen (LCA). Multi-disciplinary board meeting recommended starting neoadjuvant chemotherapy where the patient received 6 cycles of Taxol/carboplatin regimen, after which follow-up CT showed modest response with decreased size of the axillary, iliac, and paraortic lymph nodes and peritoneal masses. Bilateral sonomammography revealed a complete disappearance of the axillary nodal disease. Considering the poor performance status of the patient and the radiological response in the axillary nodes, a cytoreductive surgery was planned where total abdominal hysterectomy, bilateral salping-oophrectomy, infracolic omentectomy, and sampling of enlarged iliac lymph nodes were done. The postoperative pathological assessment revealed infiltration of both ovaries by papillary serous carcinoma confirmed by being positive for IHC WT-1, CK7, and P53 and negative for CK20 with free omentum but infiltrated iliac lymph nodes and excised peritoneal nodules. The patient had a smooth postoperative course after which she was referred back to the clinical oncologist to continue her adjuvant therapy where she received three cycles of Taxol/carboplatin regimen. She was then maintained on follow-up till she was lost to follow-up after her last visit on 11 October 2017.

## Discussion

Nearly 75% of ovarian cancer cases are diagnosed at an advanced stage (FIGO III–IV) [7]. Lymph node metastasis is considered an unusual presentation of carcinoma of the ovary as it commonly spreads by intraperitoneal route, local infiltration, or both of them leading to intra-abdominal disease with ascites and/or pleural effusion [8]. Distant metastasis is reported in stage IV disease either through lymphatic route or through hematogenous dissemination to the lungs, liver, pleura, brain, and distant lymph nodes, which is considered rare in the primary presentation of ovarian carcinoma [9].

The most commonly reported sites of lymph node metastasis from ovarian cancer are para-aortic (38%), mediastinal (29%), and pelvic (19%) while less commonly involved lymph node groups are supra-clavicular(4%) and inguinal (3%) [10, 11].

Ovarian cancer metastasis to the breast is rare representing 0.03–0.6% of all breast malignancies [12]. However, isolated axillary nodal metastasis from ovarian cancer is very rare and had been reported in just a few case reports [13].

The most common causes of malignant axillary lymphadenopathy with negative mammography include lymphoma, melanoma, and lung, stomach, or ovarian carcinoma [14].

To the best of our knowledge, 27 cases of axillary lymph node metastasis from ovarian cancer have been reported in English-based literature. A summary of their characteristics is available in Table 1.

**Table 1:** Literature review of previously reported cases of metastatic ovarian carcinoma to axillary lymph nodes

Author/year	No. of cases	Age	Presentation	Pathological type	Treatment
Hockstein 1997 [15]	1	78	Synchronous	Poorly differentiated adenocarcinoma	Platinum-based chemotherapy
Orris 1999 [16]	1	63	Recurrence	Not available	Axillary dissection
Recine 2004 [12]	6	21-67	2 synchronous/4 recurrence	Serous	Chemotherapy
Ozmen 2007 [17]	2	74/38	Recurrence	Papillary serous	Surgery
Legge 2008 [18]	1	Not available	Recurrence	Not available	Not available
Skagias 2008 [10]	1	63	Recurrence	Poorly differentiated adenocarcinoma	Not available
Aydin 2008 [4]	1	47	Recurrence	Serous	Excision + chemotherapy
Sughayer 2009 [19]	1	63	Synchronous + collision mets from breast cancer	Serous	Surgery
Harrison 2010 [20]	1	47	Synchronous	Not available	Chemotherapy
Demir 2012 [21]	1	52	Recurrence	Serous	Chemotherapy
Goyal 2012 [3]	1	68	Recurrence	Serous	Excision
Choi 2014 [22]	1	68	Synchronous	Serous	Interval debulking surgery
Patel 2014 [23]	1	50	Recurrence	Serous	Not available
Sibio 2014 [24]	1	49	Synchronous	Not available	Cytoreductive surgery
Mason 2015 [6]	1	58	Synchronous	Serous	Chemotherapy
Ilhan 2016 [13]	6	50-80	Synchronous	Serous	Interval debulking in 5 patients, chemotherapy only in one patient
Eitan 2017 [25]	2	51/70	Synchronous	Serous	Interval debulking

The most commonly reported ovarian cancer subtype that metastasizes to axillary lymph nodes is the serous subtype [12] which is the subtype reported in two of our three cases.

Whenever tumors in axillary lymph nodes are found simultaneously with ovarian cancer, a diagnostic dilemma is considered. The use of immune histochemical markers may aid in diagnosis as WT-1, CK 7, and CK 20. Cytokeratin 7 and 20 are low molecular weight cytokeratins that confirm the epithelial origin of the metastasis either from the breast or from the ovary [26]. Al-Hussaini et al. reported 94.7% of ovarian serous carcinoma to be positive for WT-1 [27].

Axillary nodal metastasis from ovarian cancer can be explained by the fact that ovarian cancer commonly presents by ascites and peritoneal carcinomatosis; hence, transdiaphragmatic invasion is possible to the superior diaphragmatic lymph nodes. From these nodes, further metastasis could follow one of two pathways. The first one is the anterior to the prepericardial lymph nodes then either to the internal jugular and subclavian veins or to the subclavian lymph trunk ending eventually into axillary lymph nodes. The second is the posterior one to deep lymphatic vessels inferior to the diaphragm and superficial lymphatic vessels inferior to the umbilicus which unites forming cisterna chyli and thoracic duct ending eventually in the junction between the left subclavian and internal jugular vein [28].

## Conclusions

To conclude, physicians should be aware of this uncommon mode of metastasis in ovarian cancer cases. This could not be encountered unless there are thorough physical examination and surveillance strategies of advanced ovarian cancer patients. Multi-disciplinary discussion is crucial in the management of these cases to offer them the best suitable treatment.

## Data Availability

All data generated or analyzed during this study are included in this published article.

## Abbreviations

CA 125: Cancer antigen 125
CK7, CK20: Cytokeratin 7,20
CT: Computed tomography
ER: Estrogen receptor
FIGO: Fédération Internationale de Gynécologie et d'Obstétrique
FNAC: Fine needle aspiration cytology
IHC: Immunohistochemical stains
LCA: Leukocyte common antigen
MRI: Magnetic resonance imaging
PR: Progesterone receptor
WT-1: Wilms’ Tumor-1
